# Changes in housing and diet combined increase fecal oxyurid load in captive tokay geckos

**DOI:** 10.7717/peerj.21014

**Published:** 2026-03-30

**Authors:** Diana S. Gliga, Birgit Szabo

**Affiliations:** 1 Institute of Parasitology, University of Bern, Bern, Switzerland; 2 Department of Biology, Universiteit Gent, Gent, Belgium; 3 Division of Behavioural Ecology, University of Bern, Bern, Switzerland

**Keywords:** Husbandry, Oxyuroidea, Parasite, Reptile, Sociality, Welfare

## Abstract

Reptiles are increasingly popular as exotic pets and can suffer high mortality risks in captivity, yet research into their welfare remains limited. Commonly occurring practices such as frequent handling, changes in housing and diet could induce stress and increase parasite loads with potentially severe negative welfare outcomes. Therefore, it is crucial to understand how routine procedures may influence parasite infections. In this study, we exposed captive bred tokay geckos (*Gekko gecko*) to two stressors common in the reptile trade, cohabitation with a novel conspecific and a change in diet. We tested the effect of a diet and housing change on oxyurid egg shedding intensity in isolation as well as the combined effect of both treatments. We found that a change in diet or housing alone had no effect on fecal parasite load while both a change in diet and housing combined did increase parasite load. However, we did not notice a visible change in the lizards’ body condition, food and water uptake or defecation within the short study period. Our study shows that common changes in housing and husbandry can increase parasite load in tokay geckos. Further studies are needed to determine which other procedures (*e.g.*, confinement, transport, novel environments) affect health and when combined could severely impact reptile health.

## Introduction

Reptiles are becoming more and more popular as exotic pets around the globe. In 2022, it was estimated that 1.5 million individuals were kept in captivity in the UK alone, an increase of 60% from 0.9 million estimated in 2016 (PFMA, 2016; PFMA, 2022). Despite this large number of individuals being held in captivity, research into reptile welfare is still lacking compared to other taxonomic groups such as mammals, but research efforts into reptile welfare are rising (Doody, 2023; Hanson et al., 2025; portions of the text were previously published as part of a preprint on bioRxiv; Gliga & Szabo, 2025).

Reptiles might suffer high risk of mortality in captivity with up to 28% of animals dying within the first year after purchase, but numbers can vary depending on the reptile group (Robinson et al., 2015). This increased mortality risk may be due to capture- and transport- induced stress (*e.g.*, noise, vibration and thermal discomfort), injury in wild caught animals, insufficient knowledge regarding a species’ ecology and natural history, and owner inexperience with either wild-caught and captive-bred animals, ultimately leading to suboptimal captive conditions and husbandry (Mancera et al., 2014; Warwick, 2014; Wilkinson, 2015). Furthermore, many captive reptiles harbor internal parasites (Arabkhazaeli et al., 2018; Ellerd et al., 2022; Hallinger et al., 2019; Rataj et al., 2011) which, under inadequate management, could contribute to the increased mortality risk. Poor hygiene can cause frequent reinfections with parasites and lead to clinically relevant burdens (Pasmans et al., 2008). Furthermore, routine procedures in reptile husbandry such as transport and frequent handling, exposure to new environments and unfamiliar conspecifics represent common stressors (Langkilde & Shine, 2006; Pasmans et al., 2008) within the pet reptile trade that could negatively affect immune function (Fischer & Romero, 2019). Captivity stress together with the stress exerted by a parasite infection might lead to the infection getting out of hand (*e.g.*, Pasmans et al., 2008; O’Dwyer et al., 2020). Therefore, a better understanding of which routine procedures cause stress and exacerbate parasite infections is needed.

Oxyurids are among the most prevalent nematodes found in captive reptiles (Hallinger et al., 2018; leopard geckos—*Eublepharis macularius*; Amaral et al., 2021; bearded dragons—*Pogona vitticeps*; Schmidt-Ukaj et al., 2017). Reptile oxyurids belong mainly to the family Phayngodonidae, in which intra-intestinal auto-infection in addition to egg deposition in the peri-anal area followed by fecal-oral infection has been demonstrated (Adamson, 1994; Jacobson & Garner, 2020). The direct life cycle of this parasite and the high resistance of the environmental stages (eggs) favor the transmission in captive settings. Bedding and enrichment material are difficult to sterilize, leading to high risk of reinfection if not done carefully (Mendyk, Newton & Baumer, 2013). Oxyurid infections are usually well tolerated in healthy reptiles (Brosda, 2013) and the species-specific parasite-host relationship is believed to have indirect beneficial effects such as improved absorption of nutrients during digestion, particularly in herbivores (Brosda, 2013). However, high burdens might lead to intestinal obstruction (Schmidt-Ukaj et al., 2017), clinical signs such as loss of appetite, lethargy, and diarrhea (Guardone et al., 2024; Pasmans et al., 2008) and in extreme cases may be fatal, as demonstrated in pet tortoises (Hallinger et al., 2018). Management of parasitic diseases could be improved if owners and reptile breeders avoid stressful husbandry procedures. However, which specific procedures are stressful enough to lead to changes in parasite load is unclear.

In this study, we investigated the effect of stressors such as a change in housing (out of single- to pair-housing) and a change in diet (from crickets to cockroaches) and their combined effect on the fecal parasite output in tokay geckos (*Gekko gecko*). Both scenarios may occur commonly after acquisition of a pet reptile or trade between owners. Tokay geckos are a very popular pet species around the globe (Grossmann, 2007). They are medium sized, nocturnal, arboreal geckos that feed mostly on insects but opportunistically also feed on small vertebrate prey (Bucol & Alcala, 2013; Grossmann, 2007). They form pairs, perform biparental care and form family groups with their offspring (Grossmann, 2007). We hypothesized that the stress related to a change in housing (new environment, unfamiliar conspecific; Langkilde & Shine, 2006) and change in diet (*e.g.*, by affecting the microbiome and/ or immune function; Fekete & Kellems, 2007; Peixoto, Harkins & Nelson, 2021) would cause a change in the parasite load and predicted an increase in parasite output in feces. However, each alone might not cause enough stress within a familiar captive environment (as used in this study), and therefore, we investigated the effect of a change in diet and housing alone as well as the effect of both stressors together on the parasite output.

## Materials & Methods

### Study animals and husbandry

We collected data from 39 adult tokay geckos. Eleven females and 10 males originated from different breeders across Europe (two females from Switzerland, nine females from Czech Republic and 10 males from Germany), while 12 females and six males were bred from these individuals in captivity at our facility (Table 1). Sex was determined by the presence (male) or absence (female) of femoral pores (Grossmann, 2007) and animals were between 1 and 9 years old (Table 1). We used all available animals.

**Table 1:** Details of sex, age, weight, origin and treatment for each of the 39 individual tokay geckos that participated in this study. ID, animal identity; Pair ID, identity of the mating partner with which each individual was housed during pair housing (“-” no mating partner as animal was housed singly throughout the study); T1, weight in December 2023; T2, weight in January 2024.

**ID**	**Pair ID**	**Sex**	**Age (years)**	**Weight T1 (g)**	**Weight T2 (g)**	**Origin**	**Diet**	**Housing**
1	6	Female	9	118	119	External	No change	Change
2	24	Female	9	100	100	External	No change	Change
3	15	Male	4	120	124	External	Change	Change
4	8	Male	4	119	122	External	No change	Change
5	18	Female	4	94	104	External	Change	Change
6	1	Male	6	130	137	External	No change	Change
7	9	Female	4	95	94	External	Change	Change
8	4	Female	4	94	95	External	No change	Change
9	7	Male	4	122	127	External	Change	Change
10	11	Female	4	90	91	External	No change	Change
11	10	Male	4	118	119	External	No change	Change
12	13	Female	4	89	103	External	Change	Change
13	12	Male	4	108	110	External	Change	Change
14	21	Male	4	129	134	External	Change	Change
15	3	Female	4	98	103	External	Change	Change
16	–	Female	6	95	102	External	No change	No change
17	20	Male	4	121	124	External	Change	Change
18	5	Male	6	124	129	External	Change	Change
19	–	Female	4	94	91	External	Change	No change
20	17	Female	4	83	86	External	Change	Change
21	14	Female	4	80	95	External	Change	Change
22	43	Male	4	105	106	External	No change	Change
24	2	Male	2	117	119	Internal	No change	Change
25	41	Female	2	90	87	Internal	Change	Change
27	42	Female	2	81	87	Internal	Change	Change
28	–	Female	2	58	61	Internal	Change	No change
30	–	Female	2	91	92	Internal	Change	No change
32	37	Female	2	84	85	Internal	No change	Change
33	38	Female	2	78	75	Internal	No change	Change
35	–	Female	2	69	75	Internal	Change	No change
36	–	Female	2	88	70	Internal	No change	No change
37	32	Male	2	105	112	Internal	No change	Change
38	33	Male	2	85	89	Internal	No change	Change
39	–	Male	2	69	70	Internal	Change	No change
40	–	Female	1	58	55	Internal	No change	No change
41	25	Male	1	93	93	Internal	Change	Change
42	27	Male	1	98	104	Internal	Change	Change
43	22	Female	1	85	82	Internal	No change	Change
44	–	Female	1	69	72	Internal	No change	No change

Geckos were housed in enclosures made of rigid foam slabs (female single housing: 45L × 45B × 70H cm, males single housing: 90L × 45B × 100H cm, pair housing: 90L  × 45B × 100H cm; enclosure setup is only suitable for scientific purposes) with glass sliding doors at the front and a top made out of mesh for improved ventilation. Enclosures included a compressed cork back wall with shelters hung on the back wall (hollow cork branches cut in half), cork branches for climbing and live plants for enrichment. The ground was made of organic rainforest soil (Dragon BIO-Ground) or seedling soil (various brands) on top and expanded clay as the bottom for drainage separated by mosquito mesh. Enclosures were set up as bioactive and included isopods and earthworms, with a layer of autoclaved red oak leaves and sphagnum moss spread over the soil to provide hiding places for invertebrates. To provide opportunity for thermoregulation, a heat mat (Tropic Shop) was fixed to the left outside wall of each enclosure locally increasing the temperature by 4–5 °C. Furthermore, all enclosures were equipped with a UVB light (Exo Terra Reptile UVB 100, 25 W) which provided UVB during the light phase. Tokay geckos are nocturnal. To be able to work with them during their natural activity period, we kept them under a reversed 12h:12h photo period (light: 6 pm to 6 am, dark: 6 am to 6 pm; including simulated sunrise and sunset). Purchased individuals had been housed under the reversed photo period for 30 months at the start of this experiment. Bred individuals had lived under these conditions their whole life. Visibility was further improved by a red light (PHILIPS TL-D 36 W/15 RED) invisible to the geckos (Loew, 1994) during the dark phase. The change in light phase was accompanied by a gradual change in temperature from 31 °C during the day and 25 °C during the night simulating natural conditions. Room humidity was set to 50% but enclosure humidity increased to 100% for a short period of time by means of reverse osmosis water provided by an automatic rain system twice a day (30s every 12 h at 5 pm and 4 am each day). The lizards were kept across three rooms, on shelves (large enclosures on the bottom and small enclosures on the top).

Geckos were fed on Mondays, Wednesdays and Fridays with 25 cm long forceps. Before the start of data collection, all geckos were fed with 3–5 adult house crickets (*Acheta domesticus*). Insects were fed with cricket mix (reptile planet LDT, which provides Vitamin D and calcium), dry cat food (various brands) and fresh carrots to provide optimal diet. A water bowl (cleaned at least once a week or more often when dirty) in each enclosure provided water ad libitum to geckos.

#### Experimental set-up and procedure

At the start of the experiment, all 39 individuals were housed singly and fed with crickets. They had been in single housing for more than a year and had been feeding on crickets since arrival or birth (except when tested for food neophobia using mealworms—*Tenebrio mollitor*, silk moth larvae—*Bombyx mori,* and locusts—*Schistocerca gregaria* in addition to crickets and cockroaches: October 2021 and April/May 2022; Szabo & Ringler, 2023a; Szabo & Ringler, 2023b). We collected a fresh, moist (within 8 h of defecation; Pasmans et al., 2008) fecal sample from the enclosure floor from all individuals during single housing between the 1st and 21st of December 2023 in the mornings (8:00 am to 12:00 pm). Geckos produce a single fecal “scat” every 2–4 days (B Szabo, pers. obs., 2022). They defecate in a latrine (also called scat pile; Grossmann, 2007) and samples can be collected from these latrines. When housed in groups, often, individuals defecate in the same latrine, although not necessarily on the same day (B Szabo, pers. obs., 2022).

At end of December 2023 (after the first fecal samples was taken), we formed 15 stable pairs moving females into the larger enclosures of males. The remaining nine individuals (eight females and one male) were kept singly for the rest of the experiment. One week after pair formation, we changed the diet of eight randomly selected pairs (using the sample function in base R to assign animals within rooms) to cockroaches (*Nauphoeta cinerea*). The reason why we waited a week after pair formation was to ensure pairs were stable and did not show any aggression towards each other. Simultaneously, we switched five randomly selected individuals that were still in single housing to a diet of cockroaches. All rooms contained animals from each treatment group. The remaining individuals were maintained on the cricket diet. Another 2–3 weeks later, we collected a second fecal sample from the enclosure floor of all individuals between the 10th and 30th of January 2024. Consequently, parasite load of single housed individuals was measured 2–3 weeks after the stressor (diet change). In the paired individuals that received a second stressor (diet change) the parasite load was measured on average two weeks later, while in the subset that did not receive a diet change, parasite load was measured on average three weeks after the housing change. No estimates of life cycle duration were available for reptile oxyurids; however, since oxyurids in other host species (*e.g.*, rodents) complete their life cycle in 7 to 25 days depending on oxyurid species we estimated that 2–3 weeks would be appropriate to measure the effect of our treatment (Pritchett-Corning & Clifford, 2012). In some cases, we were able to sample feces separately from both individuals in a pair (sharing an enclosure) but for some we were unable to determine which sample came from which individual. These were pooled into one sample (two pieces of feces) per pair. Before sample collection, all tubes were treated with UV for 15 min. All samples were collected by the same researcher (BS) who wore new gloves to collect each sample by hand (easier to grab). All samples were frozen at −20 °C, 5–10 min after collection.

#### Preliminary parasitological examination

A preliminary screening was done before the experiment on three pools of fresh fecal samples to check for the presence of parasites: (1) Fecal smears were examined natively on a glass slide with a drop of saline, (2) Ziel-Neelsen staining was conducted, and (3) the combined sedimentation-flotation method was also applied (Deplazes et al., 2016). Native smears revealed few trophozoites of flagellates. No acid-fast parasite stages were seen (*e.g.*, *Cryptosporidium* sp). The flotation slides were positive for oxyurids. DNA was extracted directly from feces using the Quick-DNA™ Fecal/Soil Microbe MiniPrep Kit (Zymo Research, USA), according to manufacturer instructions. To characterize the flagellates, a PCR that amplified the 5.8S rRNA region flanked by ITS1 and ITS2 was performed using the published ITSF and ITSR primers (Smejkalová et al., 2012). The amplicons were sequenced by an external Sanger platform (Microsynth AG, Balgach, Switzerland). The obtained sequence matched *Simplicimonas simlis* in one direction (consensus length 145 bp, 99.3% pairwise identity with KJ101561).

#### Quantitative parasitological examination

We expressed the load of parasites as oxyurid eggs per pellet of feces. Each fecal pellet was weighed and placed in a 10 ml glass tube. The tube was filled with water up halfway and vortexed. The homogenate was drained through a double gauze placed on a funnel in another glass tube. The tube was centrifuged at 600 g for 3 min. The supernatant was discarded, and a flotation solution (44% zinc chloride, density 1.320) was added with a pipetting bottle until halfway full. The sediment was loosened and mixed with a single use plastic Pasteur pipette. More flotation liquid was added until the small cupola was formed. An 18 mm × 18 mm coverslip was placed on the top of the formed cupola. The tube was centrifuged at 600 g for 5 min. Afterwards the coverslip was placed on a microscope slide and the oxyurid eggs were counted. For convenience, we used a combination of estimation and exhaustive counting. We scored the oxyurid eggs using four levels based on the number of eggs observed under the coverslip at 100× total magnification and counted using a hand tally counter (parasite score; 0 = no oxyurid eggs, 1 = 1 − 200 eggs, 2 = 201 − 300 eggs, 3 = >300 eggs). The estimation was done based on the position on the coverslip relative to its length (*e.g.*, if ≤100 eggs were counted by $ \frac{3}{4} $ of the coverslips length, we estimated <200 eggs). Animal allocation to the different treatment groups and oxyurid scoring were done by different researchers, with the individual responsible for the scoring (DG) being blind to the geckos’ condition.

#### Statistical analyses

All statistical analyses were run in R version 4.4.2 (R Core Team, 2025) and no data were excluded. We used cumulative link (mixed) models (CLM; Christensen, 2023) to analyze (1) the effect of diet (cricket and cockroach), (2) the effect of housing (single and pair housing), and (3) their combined effect on the parasite score (response variable) using subsets of data. Furthermore, we analyzed (4) the relationship of sex, age (in years) and fecal mass (to account for differences in pellet size; fixed effects) on the parasite score (response variable) before any treatment was administered. To analyze the effect of diet (1), we focused on those individuals that experienced a change in diet but not in housing (*N* = 9 single housed individuals, *N* = 4 fed crickets and *N* = 5 changed to feed on cockroaches). To analyze the effect of housing (2), we focused on the subset of individuals that experienced a housing change but no diet change (*N* = 10 individuals with repeated measures before and after the change). Finally, to analyze the combined effect of housing and diet (3), we focused on geckos in pair housing who did or did not experience a diet change (*N* = 15). Because we were unable to identify individual fecal pellets in 10 out of 15 pairs during pair housing, we calculated pair fecal mass (summing up the mass of the pellet of the male and the female) and pair parasite score (rounded up average of the individual scores). Pair parasite scores were the same for both individuals in a pair, and we used only a single score per pair to avoid pseudoreplication. To account for repeated measures in (2) we used animal identity as the random effect. No other models included random effects. We did not investigate changes in weight due to parasite infection because weight loss is slow in reptiles (B Szabo, pers. obs., 2023, also see Moore et al., 2007) and our study was likely too short to detect any noticeable change. Furthermore, from experience, many factors (*e.g.*, egg laying in females, better/worse feeding rate under different housing conditions, continuing growth of younger individuals) can have a much larger influence on weight fluctuations than the parasite infection might have had in the current study. We report the results of our analysis following the recommendations by Muff et al. (2022): *p* > 0.1 no evidence, 0.1 < *p* < 0.05 weak evidence, 0.05 < *p* < 0.01 moderate evidence, 0.01 < *p* < 0.001 strong evidence, *p* < 0.001 very strong evidence.

#### Ethical note

No invasive procedures were used to collect fecal samples. All fecal samples were collected from the enclosure floor. No animals were harmed or euthanized, and all animals were retained in our permanent colony. Humane endpoints were set to be lethargy, respiratory distress, and inability to reach food and water but were not met during this study. Captive conditions and breeding were approved by the Swiss Federal Food Safety and Veterinary Office (Laboratory animal husbandry license: No. BE4/2022). During pair formation, we monitored adults closely and immediately separated them if any aggression occurred within the first 24 h of pairing.

## Results

**Figure 1:**
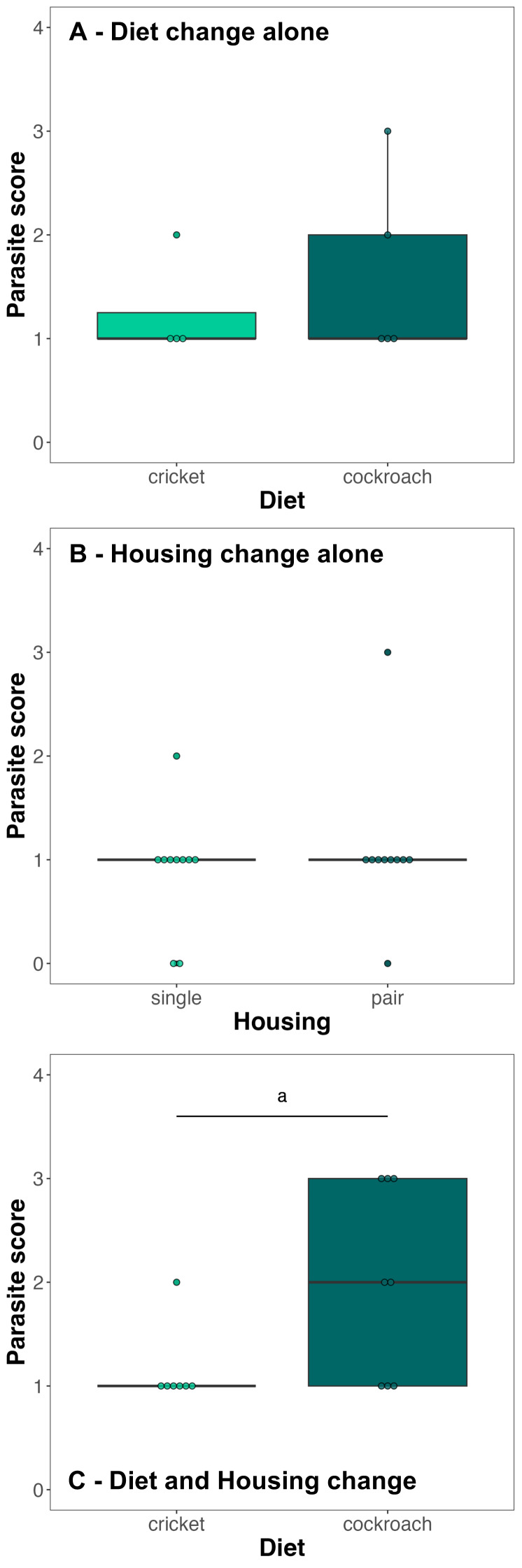
Boxplots of parasite score for animals that received only a diet change (A), only a housing change (B), or both (C). Parasite score was measured as: 0 = not detectable, 1 = 1–200 eggs, 2 = 201–300 eggs, 3 = more than 300 eggs. *N* = 9 individuals were included in (A), *N* = 10 individuals were included in (B) and *N* = 15 pairs (only one data point per pair) were included in (C). Points represent individual scores. The bold line within boxes is the median, the upper box edges are the upper quartile, the lower box edges the lower quartile, the top whisker ends are the maximum and the bottom whisker ends the minimum. a—weak evidence for a difference of *p* = 0.057.

We found no evidence that a change in diet alone predicted parasite score (CLM, estimate_no change_ = −0.888, std. error = 1.453, *z*-value = −0.611, *p*-value = 0.541; Fig. 1A). We found no evidence that a change in housing alone predicted parasite score (CLM, estimate_after change_ = 1.999, std. error = 1.970, *z*-value = 1.015, *p*-value = 0.310; Fig. 1B). We found weak evidence that a diet and housing change together predicted parasite score; individuals that received both changes had higher parasite scores (CLM, estimate_no change_ = −2.467, std. error = 1.297, *z*-value = −1.902, *p*-value = 0.057; Fig. 1C).

The overall median parasite score was 1 (IQR: 1-2). We found weak evidence that parasite score differed between males and females (CLM, estimate_male_ = 1.210, std. error = 0.722, *z*-value = 1.675, *p*-value = 0.094) and that it was associated with the mass of the fecal pellet (CLM, estimate = 2.346, std. error = 1.203, *z*-value = 1.950, *p*-value = 0.051) before any treatment was administered. We also found strong evidence that younger individuals had higher parasite loads than older individuals (CLM, estimate = −0.570, std. error = 0.220, *z*-value = −2.589, *p*-value = 0.009).

## Discussion

The aim of this study was to investigate if a change in diet and housing would impact the parasite output of captive bred tokay geckos. We found that a diet change without a change in housing or a housing change without a change in diet did not significantly impact the oxyurid output in the gecko feces. However, we found that a combination of both increased the oxyurid output which partly confirmed our hypothesis that a single stressor was not enough to influence parasite output.

In the pet trade, reptiles often experience a number of potential stressful events including capture, transport, a new housing environment and new diet (Warwick, 2014). In isolation, these events might not necessarily have a strong impact on an individuals’ health, especially when the event is short, or animals can appropriately deal with the stressor by hiding or avoidance (Broom, Johnson & Broom, 1993). However, chronic stress and/or additive stressors are more detrimental to health as opposed to isolated stressful events (Broom, Johnson & Broom, 1993), as reported in amphibians (Sih, Bell & Kerby, 2004). We found that both a change in housing and diet together increase oxyurid load. Despite this, we did not observe any noticeable decrease in the lizards’ body condition (Table 1) which would be expected if the parasite load negatively impacted health (due to appetite loss, lethargy or diarrhea; Guardone et al., 2024; Pasmans et al., 2008). In juvenile bearded dragons (*Pogona vitticeps*), a median oxyurid egg count per gram (EPG) as high as 14,409.25 did not lead to any loss in body condition over time (Pike, Hsieh & Baling, 2023). A further endoparasite survey in pet bearded dragons found high numbers of oxyurid eggs (>1,200 EPG) both in healthy and clinical cases (Guardone et al., 2024). However, our study was short and a change in body condition might have been missed if it occurred later. Additionally, our sample sizes were small, and we might not have been able to detect weak effects both on the parasite output and weight. Nonetheless, welfare might have still been impacted if the increased oxyurid load led to other discomforts that might have shown in a change in behavior which we did not measure. Future studies could investigate how different oxyurid loads (before and after treatment or reinfection) impact lizard behavior to reveal more subtle effects on welfare.

Tokay gecko males are territorial in the wild and call to attract females to their territory (Grossmann, 2007). We aimed to mimic this behavior in captivity by introducing females to male enclosures. For males, the change in housing included the addition of an unfamiliar female, while for females, it included both an enclosure change and cohabitation with an unfamiliar male. Consequently, females might have experienced higher stress, but we were not able to reliably collect fecal samples from males and females separately within the same enclosure to analyze the effect on males and females separately. Therefore, it is possible that females suffered a higher increase in oxyurid load compared to males. If females are routinely removed from social housing, for example, to prevent continuous mating, it would be important to understand the impact of husbandry procedures on each sex separately. This should be investigated in the future. Additionally, before any treatment was administered, males and females showed weak differences in their parasite load with males having slightly higher loads. In wild slow worms (*Anguis fragilis*), a sex bias in the prevalence of a nematode, *Neoxysomatium brevicaudatum*, was observed to be dependent on the time of the year; only during the breeding season did males exhibit a higher parasite prevalence which was attributed to an immunosuppressant effect of testosterone (Brown & Symondson, 2014). Tokay geckos breed all year round in captivity (Grossmann, 2007), therefore, sex differences might not occur consistently, or the effects of testosterone might be small under such captive conditions. A more systematic investigation in males could shed light on if season affects male parasite load in captive Tokay geckos similar to wild slow worms.

Our analysis also revealed a higher parasite output in younger individuals compared to older individuals. A similar result was obtained in bearded dragons in which juveniles showed a higher positivity rate for endoparasites compared to adults although this result was not significant (Guardone et al., 2024). While not significant for nematodes such as oxyurids, Guardone and colleagues (2024) found a significantly higher positivity rate for protozoa and coccidia in juveniles (70.6% *versus* 7.7% in adults). In addition, younger slow worms also exhibited higher prevenance of the nematode *Neoxysomatium brevicaudatum*. Slow worms exhibit increasing parasite infections as they age but decreasing prevalence after reaching adulthood (Brown & Symondson, 2014). Finally, a study in red-eared sliders (*Trachemys scripta*) revealed a decrease in intestinal parasite concentration with increasing plastron length (a proxy for age; Stromsland & Zimmerman, 2017). Younger individuals might have an immature immune system which could make them less capable of fighting off a parasite infection (Stromsland & Zimmerman, 2017). Nevertheless, this higher parasite output in younger individuals did not contribute to our main result as more older individuals were included in the treatment group that received both a change in housing and diet and showed increased parasite output.

In the presented husbandry our geckos appeared healthy, despite carrying oxyurids at varying intensities. Internal parasites such as nematodes are common in both wild and captive reptiles but often at a host-parasite equilibrium (Fernandes Grego, Gardiner & Catão Dias, 2004; Pasmans et al., 2008). Only if this equilibrium is disturbed by, for example anthropogenic stressors, will animals experience negative effects (Fernandes Grego, Gardiner & Catão Dias, 2004; Beldomenico & Begon, 2016). Therefore, under unfavorable husbandry conditions such parasites may lead to health issues and mortality (Pasmans et al., 2008). The presence of the detected parasites did not affect the health status of our animals even after being exposed to stressors. Therefore, our housing systems might have been able to buffer the negative effect of stressors, although we acknowledge that the size of the enclosures used in this study are only suitable for scientific purposes and larger enclosures should be used outside the scientific context.

In our study, we ensured that sample collection was done systematically and as standardized as possible. For example, we made sure that the fecal sample was collected within 8 h of defecation and only collected samples that were still fresh (Pasmans et al., 2008). Furthermore, we collected all samples in the mornings and ensured no cross contamination occurred. A previous study in bearded dragons showed that captive juveniles have 2.5x higher egg output in the afternoon than in the morning (Pike, Hsieh & Baling, 2023). Whether this is also true in tokay geckos needs to be investigated in the future. Additionally, for future studies, a more sensitive counting method, *e.g.*, using a counting chamber with a grid, could be used to obtain exact egg counts. But this would first require standardisation for very small fecal samples (<2 g).

Finally, based on molecular data we confirmed the presence of *Simplicimonas simlis* in tokay geckos. Its presence has only been reported in one other gecko species, the lined flat-tail gecko (*Uroplatus lineatus*; Čepička, Hampl & Kulda, 2010) and a ruminant (carabao, *Bubalus bubalis kerabau*; Dimasuay, Lavilla & Rivera, 2013). Its clinical relevance is not known. Further study should explore the reptile intestinal parasitome and the delicate relationship between host and facultative parasites.

## Conclusions

In conclusion, we hypothesized that seemingly small changes in housing and husbandry would affect the parasite output in captive tokay gecko. We found that only both stressors together (a change in housing and diet) led to an increase in oxyurid output. However, the general health of the animals was not visibly impacted. Future studies could examine how other routine husbandry procedures influence oxyurid output and if the stress caused by parasite infections changes behaviour in this and other species.

##  Supplemental Information

10.7717/peerj.21014/supp-1 Supplemental Information 1 ARRIVE 2.0 Checklist Checklist including the section headings and line numbers for all the requested information

## Additional Information and Declarations

### Data Availability

The following information was supplied regarding data availability: All data generated during this study and the code for analysis are available on the Open Science Framework: Szabo, Birgit, and diana gliga. 2025. “Effects of Housing and Diet on Parasite Load in a Gecko.” OSF. December 29. doi: 10.17605/OSF.IO/XJYFU.
